# Maternal exercise before and during pregnancy does not impact offspring exercise or body composition in mice

**DOI:** 10.1186/s12952-015-0032-x

**Published:** 2015-08-03

**Authors:** Scott A. Kelly, Kunjie Hua, Jennifer N. Wallace, Sarah E. Wells, Derrick L. Nehrenberg, Daniel Pomp

**Affiliations:** Department of Zoology, Ohio Wesleyan University, Schimmel/Conrades Science Center #346, 61 S. Sandusky St, Delaware, OH 43015 USA; Department of Genetics, University of North Carolina, Chapel Hill, NC 27599-7264 USA

**Keywords:** Body weight, Environment, Gestation, Pregnancy, Voluntary wheel running

## Abstract

**Background:**

The genome, the environment, and their interactions simultaneously regulate complex traits such as body composition and voluntary exercise levels. One such environmental influence is the maternal milieu (i.e., in utero environment or maternal care). Variability in the maternal environment may directly impact the mother, and simultaneously has the potential to influence the physiology and/or behavior of offspring in utero, post birth, and into adulthood. Here, we utilized a murine model to examine the effects of the maternal environment in regard to voluntary exercise (absence of wheel running, wheel running prior to gestation, and wheel running prior to and throughout gestation) on offspring weight and body composition (% fat tissue and % lean tissue) throughout development (~3 to ~9 weeks of age). Additionally, we examined the effects of ~6 weeks of maternal exercise (prior to and during gestation) on offspring exercise levels at ~9 weeks of age.

**Results:**

We observed no substantial effects of maternal exercise on subsequent male or female offspring body composition throughout development, or on the propensity of offspring to engage in voluntary wheel running. At the level of the individual, correlational analyses revealed some statistically significant relationships between maternal and offspring exercise levels, likely reflecting previously known heritability estimates for such traits.

**Conclusions:**

The current results conflict with previous findings in human and mouse models demonstrating that maternal exercise has the potential to alter offspring phenotypes. We discuss our negative findings in the context of the timing of the maternal exercise and the level of biological organization of the examined phenotypes within the offspring.

**Electronic supplementary material:**

The online version of this article (doi:10.1186/s12952-015-0032-x) contains supplementary material, which is available to authorized users.

## Background

Uncovering the factors contributing to variation in exercise participation is important from a human health perspective, as physical activity has been shown to be an effective intervention on mortality outcomes (e.g., secondary prevention of coronary heart disease, rehabilitation of stroke, treatment of heart failure, prevention of diabetes) [29]. Additionally, physical activity potentially plays a role in weight management and influences obesity risk; the dysregulation of the former and elevation of the later have both been linked to negative health outcomes. However, metabolic effects of exercise are highly variable and in part attributed to the frequency, duration, and intensity of physical activity [31], although the efficacy of exercise remains debated [5, 14, 17, 25, 26, 36, 37]. Regardless, complex traits such as body weight and voluntary exercise levels are highly variable, heritable, influenced by potentially hundreds of genes and interactions among them, and affected by the environment and its interactions with the genetic architecture [21]. One such environmental influence is the maternal environment [16].

The maternal environment is typically defined as occurring any time after the formation of the zygote and persisting through weaning (i.e., in utero environment or maternal care). More broadly, the maternal environment also likely encompasses effects on the unfertilized egg, which in turn influence offspring phenotypes post fertilization and throughout development [15]. Alterations in maternal environmental factors not only influence physiology of the mother [1], but have also been shown to impact physiology of the offspring in utero, post birth, and into adulthood [6, 12]. Moreover, influences of maternal environment may be modulated by genetic architecture of the offspring [19]. In addition to influencing the immediate offspring, maternal environment has also been shown to have intergenerational effects modulated through transgenerationally inherited epigenetic mechanisms [6, 7, 13, 23]. Notably, although not the focus of the current investigation, paternal environment (experienced across the entire lifespan) has been shown to influence offspring phenotypes in both rats and mice [30, 33].

The maternal environment may be composed of many abiotic (e.g., physical activity) and biotic (e.g., diet) factors. Recent investigations have focused on variation in maternal diet [2] and exercise [9] and the resulting impact on a variety of offspring phenotypes broadly related to the physiological control of weight, body composition, and metabolism. For example, maternal high-fat diet/obesity alters hippocampal gene expression (e.g., *N*-methyl-D-aspartate receptor (NMDA) receptor subunit NR2B as well as synaptophysin), learning, and memory function in rat offspring [32], modifies cellular development in fetal brains of rat offspring [34], induces epigenetic modifications and enhances adipogenic differentiation in mouse offspring [38], and is associated with increased childhood body weight [24]. With regard to physical activity, studies have observed that maternal exercise at various stages (prior to gestation, during gestation, during lactation) affects growth-factor expression and transiently increases postnatal hippocampal neurogenesis in mouse offspring [4], improves glucose homeostasis in adult mouse offspring [8], and improves insulin sensitivity in rat offspring [9]. Additionally, Laker et al. [22] observed that exercise (voluntary wheel running) prevented maternal high-fat diet-induced hypermethylation of *Pgc-1α* and age-dependent metabolic dysfunction in mouse offspring. In humans, exercise during pregnancy has been associated with decreased fat, improved stress tolerance, and advanced neurobehavioral maturation in offspring [10]. However, results appear to be often dependent upon the specific period of gestation when the exercise occurred [18].

Previously, we documented parent-of-origin effects on voluntary exercise levels and body composition in a fourth generation (G_4_) advanced intercross population of mice [20]. G_4_ individuals descended from progenitor (F_0_) crosses of dams selectively bred for high voluntary wheel running (HR mice) [35] and C57BL/6 J sires ran greater distances, spent more time running, ran at higher maximum speeds/ day, and had lower percent body fat and higher percent lean mass than mice descended from reciprocal progenitor crosses (C57BL/6 J♂ X HR♀). We hypothesized that maternal environment was one potential mechanism through which these parent-of-origin effects may be modulated. In the previous investigation [20] we had no direct measures of maternal activity or care, but Malisch et al. [27, 28] demonstrated that female and male HR mice exhibit an ~200 % increase in home-cage activity (in the absence of a wheel) compared with control lines.

In this report, we use a murine model to examine the effects of the absence of wheel running, wheel running prior to gestation, and wheel running prior to and throughout gestation on subsequent offspring weight and body composition (% fat tissue and % lean tissue) throughout development (~3 to ~9 weeks of age). Additionally, we examined the effects of ~6 weeks of maternal exercise (prior to and during gestation) on offspring voluntary wheel running levels at ~9 weeks of age. Moreover, we examined correlations between maternal exercise parameters (daily running distance, time spent running, average running speed, and maximum running speed) and offspring phenotypes (weight, body composition, and physical activity traits). Since we collected offspring phenotypes between the ages of ~3 and ~9 weeks, we were also able to determine if age-specific weight or body composition correlated with maternal traits throughout early development and into adulthood.

## Results

Descriptive statistics for mean running traits of the maternal generation (G_1_) are presented in Table 1. Separate sex analyses of offspring (G_2_) mean voluntary-running traits from days 5 and 6 of a 6-day test revealed no statistically significant effects of group [maternal exercise condition (none, post-weaning only, post-weaning and gestational)] on running distance (revolutions/day; *p* = 0.153, males; *p* = 0.795, females), running time (1-min intervals/day; *p* = 0.178, males; *p* = 0.853, females), average running speed (rpm; *p* = 0.204, males; *p* = 0.558, females), or maximum running speed (highest number of revolutions in any 1-min interval within a 24 h period; *p* = 0.294, males; *p* = 0.510, females) (Table 2). Estimated marginal means and standard errors corresponding to analyses of voluntary-running traits are presented in Additional file 1: Table S1.

**Table 1:** Descriptive statistics for mean running traits measured in the maternal (G_1_) population

Trait^a^	n	Mean	SD	Range	Trait^a^	n	Mean	SD	Range
*Revolutions*					*Average speed*				
~4 weeks of age	30	2,066	869	171-3,787	~4 weeks of age	28	8.6	1.4	5.8-11.3
~5 weeks of age	30	5,103	1,831	2,142-10,019	~5 weeks of age	30	13.0	2.6	8.1-21.1
~6 weeks of age	30	6,903	2,101	3,263-11,670	~6 weeks of age	30	15.4	3.9	8.7-27.4
~7 weeks of age	30	8,588	3,210	2,618-17,332	~7 weeks of age	29	17.2	5.0	8.1-33.7
~8 weeks of age	30	9,360	3,251	3,653-15,774	~8 weeks of age	30	18.1	4.8	9.0-31.6
Mating^b^	15	8,505	2,580	1,899-11,406	Mating^b^	15	13.5	2.6	6.9-15.8
Gestation^c^	14	4,079	1,085	3,021-7,119	Gestation^c^	14	10.0	2.6	6.3-15.6
*Time*					*Maximum speed*				
~4 weeks of age	30	221	82	35-329	~4 weeks of age	30	17.1	3.1	6.7-23.7
~5 weeks of age	30	375	100	159-557	~5 weeks of age	30	25.1	4.5	18.4-38.8
~6 weeks of age	30	440	89	253-633	~6 weeks of age	30	28.9	6.1	20.2-46.6
~7 weeks of age	30	476	104	173-696	~7 weeks of age	30	31.5	7.1	21.4-52.9
~8 weeks of age	30	506	94	279-696	~8 weeks of age	30	33.6	7.2	22.9-51.9
Mating^b^	15	590	159	158-793	Mating^b^	15	28.7	3.5	19.4-33.2
Gestation^c^	14	404	76	291-516	Gestation^c^	14	27.5	3.4	23.7-36.0

^a^ Traits measured as a result of exposure to running wheels: running distance (revolutions/day), time spent running (i.e., cumulative 1-min intervals in which at least one revolution was recorded), average speed (total revolutions / time spent running), and maximum speed (highest number of revolutions in any one-minute interval

^b^ Mean running traits across five days (43–47) during mating. Throughout mating, males and females both had access to the running wheel and each contributed to the total

^c^ Mean running traits across seven days (48–54) of the gestation period. Days 48–54 occurred after a confirmed pregnancy (presence of vaginal plugs) and removal of the male, but prior to giving birth. Running wheel circumference was 1.1 m

**Table 2:** Separate-sex analyses of offspring (G_2_) mean voluntary-running traits from days 5 and 6 of a 6-day exposure to running wheels

Trait^a^		Trans^b^	n	Group^c^	Age^d^	Freeness^e^
Revolutions/day	♂	none	44	*F*_2, 39_ = 1.972	*F*_1, 39_ = 0.240	*F*_1, 39_ = 0.096
				*p* = 0.153	*p* = 0.627	*p* = 0.759
	♀	log_10_	43	*F*_2, 38_ = 0.230	*F*_1, 38_ = 0.222	*F*_1, 38_ = 0.755
				*p* = 0.795	*p* = 0.640	*p* = 0.390
1-min intervals/day	♂	none	44	*F*_2, 39_ = 1.807	*F*_1, 39_ = 0.024	*F*_1, 39_ = 0.113
				*p* = 0.178	*p* = 0.877	*p* = 0.738
	♀	none	43	*F*_2, 38_ = 0.159	*F*_1, 38_ = 0.014	*F*_1, 38_ = 2.612
				*p* = 0.853	*p* = 0.905	*p* = 0.114
Average speed (rpm)	♂	log_10_	44	*F*_2, 39_ = 1.658	*F*_1, 39_ = 2.834	*F*_1, 39_ = 2.621
				*p* = 0.204	*p* = 0.100	*p* = 0.113
	♀	log_10_	43	*F*_2, 38_ = 0.593	*F*_1, 38_ = 0.261	*F*_1, 38_ = 7.254
				*p* = 0.558	*p* = 0.613	*p* = 0.010
Maximum speed (rpm)	♂	log_10_	44	*F*_2, 39_ = 1.263	*F*_1, 39_ = 0.054	*F*_1, 39_ = 1.096
				*p* = 0.294	*p* = 0.818	*p* = 0.302
	♀	log_10_	43	*F*_2, 38_ = 0.686	*F*_1, 38_ = 0.612	*F*_1, 38_ = 7.299
				*p* = 0.510	*p* = 0.439	*p* = 0.010

^a^ Revolutions/day (distance), 1-min intervals/day (time, cumulative 1-min intervals in which at least one revolution was recorded), average running speed (total revolutions/time spent running), and maximum running speed (highest number of revolutions in any 1-min interval within a 24 h period). Data were from general linear model [Univariate GLM ANOVA (SPSS, Chicago, IL)] and transformed

^b^ as necessary to improve normality of residuals [11]

^c^ Group represents the following maternal experimental conditions: no exercise (standard mouse cage), post-weaning only exercise (access to a running wheel up until the time of mating), or post-weaning and gestational exercise (access to a running wheel until two days prior to giving birth). Additionally, age

^d^ and wheel freeness

^e^ (number of wheel revolutions following acceleration to a given velocity) were included in the model as covariates. Significance levels (*P*-values: bold indicates *p* < 0.05) for the effects of maternal (G_1_) exercise group (none, post-weaning only, post-weaning and gestational). The following covariates were also included in the analyses: Age, days since birth at the time of initial exposure to running wheel; Freeness, number of wheel revolutions following acceleration to a given velocity. Running wheel circumference was 1.1 m

**Fig. 1:**
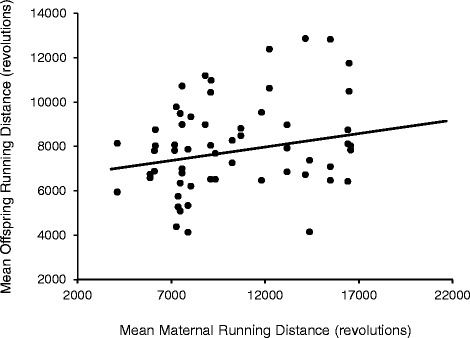
Relationship between mean offspring (G_2_) running distance (revolutions/day) and mean maternal (G_1_) running distance. G_2_ values represent the means of days 5 and 6 of a 6-day wheel exposure at ~9 weeks of age. G_1_ values are the averages of days 33 and 34 of wheel access. The G_1_ running trait values are approximately age matched to the values of the offspring. Pearson partial correlations (*r*; controlling for sex) revealed a statically significant relationship between the two running variables (*p* = 0.031, *r* = 0.282)

**Fig. 2:**
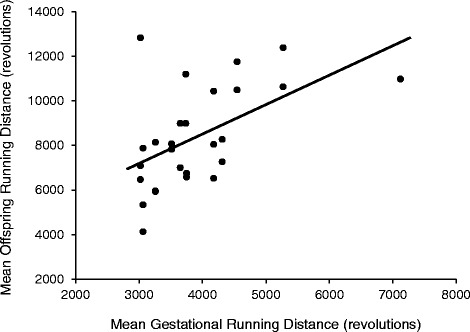
Relationship between mean offspring (G_2_) running distance (revolutions/day) and mean gestational (G_1_) running distance. G_2_ values represent the means of days 5 and 6 of a 6-day wheel exposure at ~9 weeks of age. G_1_ running trait values are the means of days 48–54 of wheel access. Days 48–54 occurred after a confirmed pregnancy (presence of vaginal plugs) and removal of the male, but prior to giving birth. Pearson partial correlations (*r*; controlling for sex) revealed a statically significant relationship between the two running variables (*p* = 0.001, *r* = 0.600)

At the level of the individual, Pearson partial correlations (*r*, controlling for sex) revealed that mean offspring-running distance was statistically significantly correlated with maternal mean running distance (*p* = 0.031, *r* = 0.282, Fig. 1) and running time (*p* = 0.039, *r* = 0.269) prior to gestation (Additional file 1: Table S2). Additionally, offspring (G_2_) running time was statistically significant correlated to maternal (G_1_) running time prior to gestation (*p* = 0.004, *r* = 0.371) (Additional file 1: Table S2). Maternal values utilized for correlations represented the averages of days 33 and 34 of wheel access, so as to be approximately age matched with values of the offspring. Pearson partial correlations (controlling for sex) were also performed for maternal-gestational (G_1_) and offspring (G_2_) mean voluntary running traits (Additional file 1: Table S3). G_1_ running trait values are the means of days 48–54 of wheel access. Days 48–54 occurred after a confirmed pregnancy (presence of vaginal plugs) and removal of the male, but prior to giving birth. Correlations revealed a statistically significant relationship between mean offspring running distance and maternal-gestational running distance (*p* = 0.001, *r* = 0.600; Fig. 2) and maximum running speed (*p* = 0.046, *r* = 0.395). Additionally, average and maximum running speeds of offspring were statistically significantly correlated with maternal maximum running speeds during gestation (*p* = 0.009, *r* = 0.501; *p* = 0.013, *r* = 0.482; respectively). These mother-offspring correlations likely represent rough estimates of heritability, and are similar to what have been previously reported in mice of similar genetic background (see Ref. [35]). For additional information on the genetic architecture underlying voluntary exercise behavior see [21] and references therein.

**Fig. 3:**
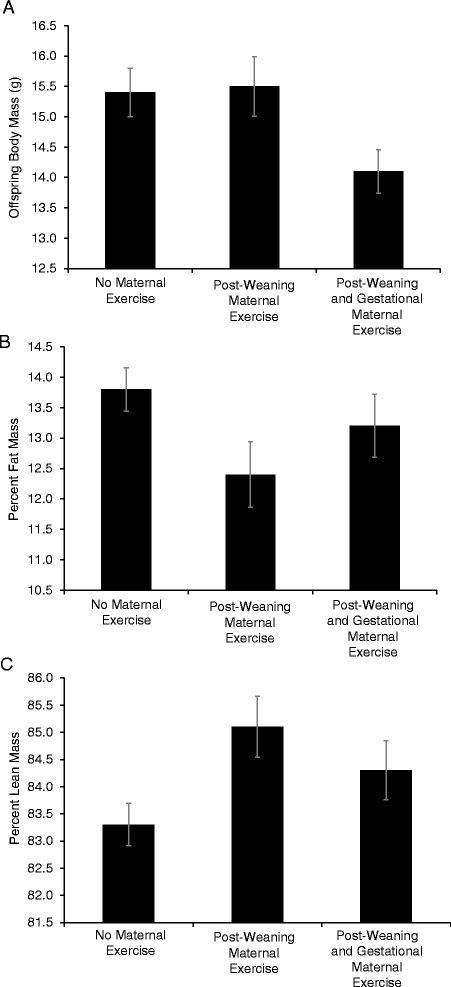
Female offspring (G_2_) body mass (**a**), percent fat mass (**b**), and percent lean mass (**c**) at 3 weeks of age. Individuals were exposed to the following maternal experimental conditions: no maternal exercise (standard mouse cage), post-weaning maternal exercise (access to a running wheel up until the time of mating), and post-weaning and gestational maternal exercise (access to a running wheel until two days prior to giving birth). General Linear Models [Univariate GLM ANOVA (SPSS, Chicago, IL)] revealed marginal effects of maternal exercise condition on body mass (*F*_2, 42_ = 3.089, *p* = 0.056) and percent lean mass (*F*_2, 42_ = 3.356, *p* = 0.044), but not on percent fat mass (*F*_2, 42_ = 2.289, *p* = 0.114). Means ± standard errors of untransformed data are presented for each trait

Results of separate-sex analyses of offspring (G_2_) body composition traits at different ages and in response to 6 days of voluntary wheel running are presented in Additional file 1: Table S4. Age, days since birth at the time of phenotypic measurement was included as a covariate where appropriate. Analyses revealed only one statistically significant effect of maternal exercise condition group on percent lean mass at 3 weeks of age for female offspring (*p* = 0.044) (Fig. 3). Estimated marginal means and standard errors corresponding to analyses of body composition traits are presented in Additional file 1: Table S5.

## Discussion

Variability in predisposition to engage in voluntary activity is modulated by many factors and their interactions [21]. Moreover, the environment, a known modulator of physical activity levels, is itself composed of many factors acting simultaneously to influence the propensity to engage in exercise. We previously observed parent-of-origin effects on voluntary exercise levels and body composition in a fourth generation (G_4_) advanced intercross population of mice and hypothesized that maternal environment was one potential mechanism through which these effects may be modulated [20]. Here, we made direct measures of maternal (G_1_) activity prior to and throughout gestation, but found no evidence that offspring (G_2_) body composition throughout development or activity levels as adults were affected. Below, we discuss the implications of our negative findings in the context of previous investigations and suggest avenues for future research directions.

Previous human investigations have demonstrated that effects of maternal exercise on offspring phenotypes are dependent on the specific period of gestation when the activity occurred [18]. In the current experiment, we observed a sharp decline in voluntary wheel running associated with the onset and progression of gestation, but wheel-running values did not completely regress to zero (see Table 1). That is, regardless of the stage of gestation, females granted wheel access were presumed to be more active than those that were housed without a running wheel (we did not monitor the home cage activity of the G_1_ females housed in the absence of a running wheel). Therefore, although we view it as unlikely, it is possible that the G_1_ females not granted wheel access were equally active in their home cages as G_1_ females with wheel access. We do acknowledge that although G_1_ females engaged in substantial activity at all time points, the running volume may have been insufficient to garner an observable response in the phenotypes measured in the offspring. However, the running values we observed prior to and following gestation were similar to what has been previously been shown to elicit a phenotypic response in offspring of the same strain of mice (ICR) [8].

Carter et al. [8] granted mice access (or not) to a running wheel prior to mating and during gestation and nursing, and then measured their offspring. These researchers observed that maternal exercise improved offspring glucose disposal following an oral glucose challenge, reduced blood glucose concentrations following an intraperitoneal insulin tolerance test in offspring, and in male offspring increased percent lean mass and decreased percent fat mass [8]. Similar to the current study, Carter et al. [8] did not observe any changes in offspring body weight as a result of maternal exercise. Unlike the current study, the mice in Carter et al. [8] were granted wheel access during lactation, which may alter nursing patterns and/or milk nutritional content, potentially influencing offspring physiological and behavioral phenotypes. Although wheel running was substantially reduced during lactation (see Fig. 1a in Ref. [8]) the activity may have been sufficient and necessary to have an effect on offspring phenotypes. Maternal exercise effects (increased hippocampal neurogenesis in offspring) have also been observed in other studies where wheel access was available during lactation as well as gestation [4].

In addition to the importance of the “timing” of an altered maternal environment, it may also be vital to examine the potential effects at the right level of biological organization within the offspring. Here, we examined the effects of maternal exercise at relatively high levels of biological organization with the offspring (e.g., behavior, body weight). Although we observed very little impact of maternal exercise on these traits, it is possible that traits underlying these complex phenotypes (e.g., neurogenesis, glucose tolerance, insulin sensitivity, gene expression alterations in the hippocampus or hypothalamus, etc.) may have been affected. Other rodent studies, investigating lower level phenotypes (e.g., cellular, molecular, biochemical) in offspring have been quite successful in demonstrating a fairly robust effect of running during gestation and lactation [4, 8]. However, at this time we are unaware of additional murine studies that have examined the effects of maternal exercise on the propensity of offspring to engage in voluntary wheel running behavior as adults. Although we measured body composition related traits throughout development in G_2_ mice, we only measured activity levels at single time point (~9 weeks of age). Future investigations should assess physical activity levels of offspring throughout development while simultaneously using a more integrative approach, measuring offspring phenotypes at a variety of levels of biological organization temporally. Additionally, future studies should consider direct effects (physiological impacts of the mother exercising on the developing fetus) *versus* epigenetic and transgenerational epigenetic influences.

## Conclusions

In the present study we observed no substantial effects of maternal exercise on subsequent male or female offspring body composition throughout development, or on the propensity of offspring to engage in voluntary wheel running. At the level of the individual, correlational analyses revealed some statistically significant relationships between maternal and offspring exercise levels, likely reflecting previously known heritability estimates for such traits. The current results conflict with previous findings in human and mouse models demonstrating that maternal exercise has the potential to alter offspring phenotypes. However, as discussed above, our negative findings may be a reflection of the examination of the effects of maternal exercise at relatively high levels of biological organization (e.g., behavior, body weight) as opposed to traits underlying these complex phenotypes. Additionally, we encourage future investigations to examine offspring activity levels over the course of development as opposed to only at adulthood as was done here.

## Methods

### Generation 0 – base population animals

All procedures were approved by and are in accordance with guidelines set forth by the Institutional Animal Care and Use Committee at The University of North Carolina at Chapel Hill. At the conclusion of the current study, all mice utilized were euthanized in accordance with guidelines set forth by the Institutional Animal Care and Use Committee at The University of North Carolina at Chapel Hill. Hsd: ICR [Harlan Sprague–Dawley (HSD): Institute of Cancer Research (ICR)] mice (*n* = 17, females, ~7 weeks of age; *n* = 17, males, ~11 weeks of age) were obtained from Harlan Laboratories (Indianapolis, IN). Hsd: ICR mice were utilized because they served as one of the founding strains of an advanced intercross population in which we previously observed parent-of-origin effects [3, 20]. In [20] we hypothesized that the parent-of-origin effects may have been a result of an altered maternal environment (i.e., higher levels of physical activity). As the maternal effects may be strain specific, we chose to utilize Hsd: ICR mice in the current investigation. All mice were reared in a viral-free facility, maintained at a temperature of 22 °C and 30–55 % relative humidity, and exposed to a light–dark cycle of 12 h:12 h beginning at 0700. Food (Prolab Isopro RMH 3000; calories provided by: protein 26 %, fat 14 %, carbohydrates 60 %) and water were provided *ad libitum*. During gestation and lactation, breeding pairs were provided an enriched diet (Prolab RMH 2000; calories provided by protein 22 %, fat 23 %, carbohydrates 55 %). The base population animals (generation 0, G_0_) were paired for breeding after a two-week acclimation period. These breeding pairs were utilized to generate 15 females (generation 1, G_1_) for each of three experimental conditions (no exercise, exercise post-weaning, and exercise post weaning and gestational).

### Generation 1 – maternal phenotypes

At 3 weeks of age, female G_1_ mice were weaned, weighed, and body composition (% fat tissue and % lean tissue) measured utilizing an EchoMRI-100 quantitative magnetic resonance whole body composition analyzer (Echo Medical Systems, Houston, TX). Percent body fat (and lean) was calculated as (fat mass/body mass)*100. Following body composition measures, mice were singly housed and randomly assigned to one of three experimental conditions: 1) no exercise (*n* = 15, standard mouse cage), 2) post-weaning only exercise (*n* = 15, access to a running wheel up until the time of mating); or 3) post-weaning and gestational exercise (*n* = 15, access to a running wheel until two days prior to giving birth). In the two experimental groups with access to running wheels (model 80850, circumference = 1.1 m; Lafayette Instruments, Lafayette, IA), daily wheel-running activity was monitored with Running Wheel Activity Software (AWM V9.2, Lafayette Instruments) via Activity Wheel Counters (model 86061, Lafayette Instruments) interfaced with computers. Wheel-running activity was recorded in 1-min intervals for 23–24 h of each of the days of wheel access. From this information, the following daily traits were calculated: total daily revolutions, time spent running (i.e., cumulative 1-min intervals in which at least one revolution was recorded), average speed (total revolutions/time spent running), and maximum speed (highest number of revolutions in any 1-min interval within a 24 h period).

G_1_ mice, in all three experimental groups, were weighed, measured for body composition, and monitored for food consumption on a weekly basis. To minimize any variation in food consumption due to food wasting, bedding was examined and any visible pieces of food were accounted for. To produce a second generation (G_2_), at ~9 weeks of age, female mice from all three experimental groups were singly mated with ~11 week old Hsd: ICR males purchased from Harlan Laboratories. At this time, wheel access was blocked for experimental group 2 (*n* = 15, post-weaning only exercise). For experimental group 3 [post-weaning and gestational exercise (*n* = 15)], wheel access was continuous throughout mating and gestation (wheel access was denied two days prior to birth and through the lactation period). Following mating, all females were checked daily for the presence of vaginal plugs and once observed the male was removed from the cage. After females gave birth (postnatal day 3), where applicable, all litters were culled to 4 females and 4 males to standardize litter size.

### Generation 2 – offspring phenotypes

At 3 weeks of age, G_2_ mice were weaned, weighed, and body composition (% fat tissue and % lean tissue) measured as described above. 15 females and 15 males, randomly selected from each of the three G_1_ maternal experimental groups, were housed (4 per cage) in sex-specific cages (in total, *n* = 45 females and *n* = 45 males). The number of G_2_ litters represented from each G_1_ (maternal) group was as follows: 15, no exercise; 14, post-weaning only exercise; 13, post-weaning and gestational exercise. All mice (*n* = 90) were weighed and body composition measured weekly. At ~9 weeks of age, following weight and body composition measures, mice were individually given access to running wheels for 6 days, during which daily wheel-running activity was monitored as described above. Following the 6th day of wheel access, mice were weighed, body composition assessed, and food consumption calculated. Percent change as a result of exercise in percent body fat (and lean) was calculated as [(% following wheel access – % prior to wheel access)/% prior to wheel access]*100.

### Statistical analysis

Effects of maternal exercise condition (none, post-weaning only, post-weaning and gestational) on offspring body weight, body composition (% fat and % lean), exercise predisposition, and change in body weight and composition as a result of exercise were determined using a General Linear Model [Univariate GLM ANOVA (SPSS, Chicago, IL)]. Sexes were analyzed separately because of known differences in the phenotypes of interest. The primary grouping factor was maternal exercise condition (fixed effect). Due to the slight variation in age, it was included as a covariate in all analyses. Additionally, where applicable, body mass and wheel freeness (number of wheel revolutions following acceleration to a given velocity) were included in the model as covariates. Traits were transformed as needed to stabilize variances among groups and improve normality of residuals. Statistical significance was judged at P > 0.05, and all P-values presented are two-tailed.

## Additional file

Additional file 1: Table S1. Estimated marginal means and standard errors from SPSS, corresponding to tests (separate-sex analyses) presented in Table 1. Means represent the effects of maternal exercise condition (none, post-weaning only, post-weaning and gestational) on offspring (G_2_) exercise traits, body weight, body composition, and change in body weight and composition as a result of exercise. **Table S2.** Pearson partial correlations for maternal (G_1_) and offspring (G_2_) mean voluntary running traits. Correlations were generated with individuals (G_1_ and G_2_) representing two of three maternal exercise conditions (post-weaning only, post-weaning and gestational). **Table S3.** Pearson partial correlations for maternal-gestational (G_1_) and offspring (G_2_) mean voluntary running traits. Correlations were generated with individuals (G_1_ and G_2_) representing one of three maternal exercise conditions (post-weaning and gestational). **Table S4.** Separate-sex analyses of offspring (G_2_) body composition traits at different ages and in response to 6 days of voluntary wheel running. **Table S5.** Estimated marginal means and standard errors from SPSS, corresponding to tests (separate-sex analyses) presented in Table S4. Note that data transformation is not uniform for males and females for a given trait. Means represent the effects of maternal exercise condition (none, post-weaning only, post-weaning and gestational) on offspring (G_2_) body weight, body composition, and change in body weight and composition as a result of exercise.
