# Supplementary material for: Maternal exercise before and during pregnancy does not impact offspring exercise or body composition in mice
Source: J Negat Results Biomed. 2015 Aug 3;14:13. doi: 10.1186/s12952-015-0032-x (PMC4522962; doi:10.1186/s12952-015-0032-x)
Supplement: Additional file 1: Table S1. — Estimated marginal means and standard errors from SPSS, corresponding to tests (separate-sex analyses) presented in Table 1. Means represent the effects of maternal exercise condition (none, post-weaning only, post-weaning and gestational) on offspring (G2) exercise traits, body weight, body composition, and change in body weight and composition as a result of exercise. Table S2. Pearson partial correlations for maternal (G1) and offspring (G2) mean voluntary running traits. Correlations were generated with individuals (G1 and G2) representing two of three maternal exercise conditions (post-weaning only, post-weaning and gestational). Table S3. Pearson partial correlations for maternal-gestational (G1) and offspring (G2) mean voluntary running traits. Correlations were generated with individuals (G1 and G2) representing one of three maternal exercise conditions (post-weaning and gestational). Table S4. Separate-sex analyses of offspring (G2) body composition traits at different ages and in response to 6 days of voluntary wheel running. Table S5. Estimated marginal means and standard errors from SPSS, corresponding to tests (separate-sex analyses) presented in Table S4. Note that data transformation is not uniform for males and females for a given trait. Means represent the effects of maternal exercise condition (none, post-weaning only, post-weaning and gestational) on offspring (G2) body weight, body composition, and change in body weight and composition as a result of exercise. [file 12952_2015_32_MOESM1_ESM.pdf]

Table S1 Estimated marginal means and standard errors from SPSS, corresponding to tests (separate-sex analyses) presented in Table 1. Means represent the effects of maternal exercise condition (none, post-weaning only, post-weaning and gestational) on offspring (G_2) exercise traits, body weight, body composition, and change in body weight and composition as a result of exercise.

**Trait**[a]	**None**				**Post-weaning**				**Post-weaning & gestational**			
	Male		Female		Male		Female		Male		Female	
	Mean	SE	Mean	SE	Mean	SE	Mean	SE	Mean	SE	Mean	SE
Revolutions/day	5925.998	571.705	3.940	0.030	6977.516	569.119	3.913	0.290	7547.943	596.828	3.917	0.030
1-minute intervals/day	368.515	28.676	531.786	27.203	438.001	28.546	535.876	26.201	433.162	29.936	515.668	27.052
Average speed (rpm)	1.196	0.015	1.219	0.022	1.200	0.015	1.189	0.021	1.233	0.016	1.214	0.022
Maximum speed (rpm)	1.466	0.014	1.466	0.018	1.443	0.013	1.443	0.018	1.472	0.014	1.471	0.018

[a]Revolutions/day (distance), 1-minute intervals/day (time, cumulative 1-minute intervals in which at least one revolution was recorded), average running speed (total revolutions/time spent running), and maximum running speed (highest number of revolutions in any 1-min interval within a 24 h period).

Table S2 Pearson partial correlations for maternal (G_1) and offspring (G_2) mean voluntary running traits. Correlations were generated with individuals (G_1 and G_2) representing two of three maternal exercise conditions (post-weaning only, post-weaning and gestational).

**Trait**	G_2 Time	G_2 Average Speed	G_2 Maximum Speed	G_1 Distance	G_1 Time	G_1 Average Speed	G_1 Maximum Speed
G_2 Distance	0.654**	0.646**	0.525**	0.282*	0.269*	0.227	0.233
G_2 Time		-0.144	-0.224	0.227	0.371*	0.068	0.011
G_2 Average Speed			0.914**	0.105	-0.032	0.186	0.237
G_2 Maximum Speed				0.060	-0.102	0.178	0.252
G_1 Distance					0.727**	0.837**	0.762**
G_1 Time						0.246	0.190
G_1 Average Speed							0.918**

Pearson partial correlations (r; controlling for sex) are shown for revolutions/day (distance), 1-minute intervals/day (time, cumulative 1-minute intervals in which at least one revolution was recorded), average running speed (total revolutions/time spent running), and maximum running speed (highest number of revolutions in any 1-min interval within a 24 h period). G_1 values are the averages of days 33 and 34 of wheel access. The G_1 running trait values are approximately age matched to the values of the offspring (G_2). Running wheel circumference was 1.1 m. $^{*}p < 0.05$. $^{**}p < 0.001$.

Table S3 Pearson partial correlations for maternal-gestational (G_1) and offspring (G_2) mean voluntary running traits. Correlations were generated with individuals (G_1 and G_2) representing one of three maternal exercise conditions (post-weaning and gestational).

**Trait**	G_2 Time	G_2 Average Speed	G_2 Maximum Speed	G_1 Distance	G_1 Time	G_1 Average Speed	G_1 Maximum Speed
G_2 Distance	0.573^{**}	0.694^{**}	0.719^{**}	0.600^{**}	0.321	0.357	0.395^{*}
G_2 Time		-0.181	-0.104	0.409^{*}	0.349	0.143	-0.081
G_2 Average Speed			0.965^{**}	0.372	0.077	0.301	0.501^{*}
G_2 Maximum Speed				0.379	0.065	0.292	0.482^{*}
G_1 Distance					0.272	0.622^{*}	0.458^{*}
G_1 Time						-0.515^{*}	-0.089
G_1 Average Speed							0.594^{*}

Pearson partial correlations (r; controlling for sex) are shown for revolutions/day (distance), 1-minute intervals/day (time, cumulative 1-minute intervals in which at least one revolution was recorded), average running speed (total revolutions/time spent running), and maximum running speed (highest number of revolutions in any 1-min interval within a 24 h period). G_1 running trait values are the means of days 48-54 of wheel access. Days 48-54 occurred after a confirmed pregnancy (presence of vaginal plugs) and removal of the male, but prior to giving birth. Running wheel circumference was 1.1 m. $^{*}p < 0.05$. $^{**}p < 0.001$.

Table S4 Separate-sex analyses of offspring (G_2) body composition traits at different ages and in response to 6 days of voluntary wheel running.

Trait		Trans[c]	n	Group	Age
~3 weeks of age					
Body mass, g	‖	none	45	$F_{2, 42} = 2.409$ $p = 0.102$	
	|	none	45	$F_{2, 42} = 3.089$ $p = 0.056$	
% Fat	‖	log10	45	$F_{2, 42} = 0.301$ $p = 0.742$	
	|	^2.5	45	$F_{2, 42} = 2.289$ $p = 0.114$	
% Lean	‖	none	45	$F_{2, 42} = 0.022$ $p = 0.979$	
	|	log_{10}	45	$F_{2, 42} = 3.356$ $p =$ **0.044**	
~4 weeks of age					
Body mass, g	‖	none	44	$F_{2, 40} = 1.468$ $p = 0.243$	$F_{1, 40} = 17.317$ $p <$ **0.001**
	|	log10	44	$F_{2, 40} = 3.184$ $p = 0.052$	$F_{1, 40} = 17.761$ $p <$ **0.001**
% Fat	‖	log10	44	$F_{2, 40} = 0.042$ $p = 0.959$	$F_{1, 40} = 2.410$ $p = 0.128$
	|	none	44	$F_{2, 40} = 0.810$ $p = 0.452$	$F_{1, 40} = 0.651$ $p = 0.425$
% Lean	‖	none	44	$F_{2, 40} = 0.158$ $p = 0.855$	$F_{1, 40} = 2.405$ $p = 0.129$
	|	none	44	$F_{2, 40} = 0.325$ $p = 0.724$	$F_{1, 40} = 0.210$ $p = 0.649$
~5 weeks of age					
Body mass, g	‖	log10	43[b]	$F_{2, 39} = 0.228$ $p = 0.797$	$F_{1, 39} = 4.070$ $p = 0.051$
	|	log10	43[b]	$F_{2, 39} = 0.786$ $p = 0.463$	$F_{1, 39} = 11.282$ $p =$ **0.002**
% Fat	‖	log10	43[b]	$F_{2, 39} = 1.739$ $p = 0.189$	$F_{1, 39} = 0.046$ $p = 0.831$
	|	none	43[b]	$F_{2, 39} = 0.092$ $p = 0.912$	$F_{1, 39} = 1.239$ $p = 0.272$
% Lean	‖	none	43[b]	$F_{2, 39} = 2.387$ $p = 0.105$	$F_{1, 39} = 0.629$ $p = 0.433$
	|	none	43[b]	$F_{2, 39} = 1.338$ $p = 0.274$	$F_{1, 39} = 4.230$ $p =$ **0.046**
~6 weeks of age					
Body mass, g	‖	none	44	$F_{2, 40} = 0.046$ $p = 0.955$	$F_{1, 40} = 4.006$ $p = 0.052$
	|	log10	44	$F_{2, 40} = 0.046$ $p = 0.955$	$F_{1, 40} = 1.767$ $p = 0.191$
% Fat	‖	log10	44	$F_{2, 40} = 1.515$	$F_{1, 40} = 0.420$

				p = 0.232	p = 0.521
	|	none	44	$F_{2, 40}$ = 0.198<br>p = 0.821	$F_{1, 40}$ = 0.006<br>p = 0.938
% Lean	‖	^6.0	44	$F_{2, 40}$ = 1.218<br>p = 0.306	$F_{1, 40}$ = 1.003<br>p = 0.322
	|	none	44	$F_{2, 40}$ = 0.355<br>p = 0.703	$F_{1, 40}$ = 0.039<br>p = 0.844
~7 weeks of age					
Body mass, g	‖	log10	44	$F_{2, 40}$ = 0.214<br>p = 0.808	$F_{1, 40}$ = 4.700<br>p = **0.036**
	|	log10	44	$F_{2, 40}$ = 0.050<br>p = 0.951	$F_{1, 40}$ = 6.546<br>p = **0.014**
~8-9 weeks of age[c]					
Body mass, g	‖	none	44	$F_{2, 40}$ = 0.191<br>p = 0.827	$F_{1, 40}$ = 1.035<br>p = 0.315
	|	log10	44	$F_{2, 40}$ = 0.209<br>p = 0.812	$F_{1, 40}$ = 1.866<br>p = 0.180
% Fat	‖	log10	44	$F_{2, 40}$ = 1.763<br>p = 0.185	$F_{1, 40}$ = 1.600<br>p = 0.213
	|	none	44	$F_{2, 40}$ = 0.615<br>p = 0.546	$F_{1, 40}$ = 0.029<br>p = 0.867
% Lean	‖	^5.0	44	$F_{2, 40}$ = 1.649<br>p = 0.205	$F_{1, 40}$ = 2.143<br>p = 0.151
	|	none	44	$F_{2, 40}$ = 0.299<br>p = 0.743	$F_{1, 40}$ = 0.179<br>p = 0.674
Post exercise					
Body Mass, g	‖	^2.5	44	$F_{2, 40}$ = 0.840<br>p = 0.439	$F_{1, 40}$ = 0.005<br>p = 0.944
	|	log10	44	$F_{2, 40}$ = 0.367<br>p = 0.695	$F_{1, 40}$ = 1.227<br>p = 0.275
% Fat	‖	log10	44	$F_{2, 40}$ = 1.625<br>p = 0.210	$F_{1, 40}$ = 2.240<br>p = 0.142
	|	^0.5	44	$F_{2, 40}$ = 0.136<br>p = 0.873	$F_{1, 40}$ = 0.001<br>p = 0.971
% Lean	‖	^5.0	44	$F_{2, 40}$ = 2.178<br>p = 0.126	$F_{1, 40}$ = 5.503<br>p = **0.024**
	|	none	44	$F_{2, 40}$ = 0.139<br>p = 0.871	$F_{1, 40}$ = 0.198<br>p = 0.659
% Change in mass	‖	none	43[b]	$F_{2, 39}$ = 2.308<br>p = 0.113	$F_{1, 39}$ = 1.907<br>p = 0.175
	|	none	44	$F_{2, 40}$ = 0.032<br>p = 0.969	$F_{1, 40}$ = 0.880<br>p = 0.354
% Change in % fat	‖	none	43[b]	$F_{2, 39}$ = 0.537<br>p = 0.589	$F_{1, 39}$ = 0.604<br>p = 0.442
	|	none	44	$F_{2, 40}$ = 0.972<br>p = 0.387	$F_{1, 40}$ < 0.001<br>p = 0.998
% Change in % lean	‖	none	43[b]	$F_{2, 39}$ = 3.228<br>p = 0.050	$F_{1, 39}$ = 0.402<br>p = 0.718
	|	none	44	$F_{2, 40}$ = 0.009<br>p = 0.992	$F_{1, 40}$ = 2.245<br>p = 0.142

Data were from general linear models [Univariate GLM ANOVA (SPSS, Chicago, IL)] and transformed[a] as necessary to improve normality of residuals. Significance levels (P-values: bold indicates $p < 0.05$) for the effect of maternal (G_1) exercise group (none, post-weaning only, post-weaning and gestational). Age, days since birth at the time of phenotypic measurement was included as a covariate where appropriate. [b]Removal of one or more outliers based on formal statistical test [37,38]. [c]At ~8 weeks of age body composition measures were taken immediately prior to (in) and following (out) 6 days of wheel access. Percent body fat (and lean) was calculated as (fat mass/body mass)*100. Percent change variables were calculated as [(out - in)/in]*100.

Table S5 Estimated marginal means and standard errors from SPSS, corresponding to tests (separate-sex analyses) presented in Supplementary Table 4. Note that data transformation is not uniform for males and females for a given trait. Means represent the effects of maternal exercise condition (none, post-weaning only, post-weaning and gestational) on offspring (G_2) body weight, body composition, and change in body weight and composition as a result of exercise.

Trait	None				Post-weaning				Post-weaning & gestational			
	Male		Female		Male		Female		Male		Female	
	Mean	SE	Mean	SE	Mean	SE	Mean	SE	Mean	SE	Mean	SE
~3 weeks of age												
Body Mass, g	16.307	0.519	15.367	0.421	15.607	0.519	15.493	0.421	14.700	0.519	14.153	0.421
% Fat	1.112	0.021	732.147	55.320	1.094	0.021	564.832	55.320	1.092	0.021	653.133	55.320
% Lean	85.367	0.684	1.920	0.003	84.652	0.637	1.930	0.003	84.954	0.598	1.926	0.003
~4 weeks of age												
Body Mass, g	25.717	0.539	1.314	0.010	25.570	0.537	1.318	0.010	24.493	0.557	1.284	0.011
% Fat	3.303	0.075	13.660	0.589	3.300	0.075	12.605	0.589	3.329	0.078	13.229	0.610
% Lean	86.988	0.538	82.747	0.597	86.637	0.536	83.419	0.598	86.596	0.557	82.982	0.619
~5 weeks of age												
Body Mass, g	1.480	0.009	1.391	0.010	1.485	0.009	1.401	0.011	1.489	0.009	1.382	0.011
% Fat	1.089	0.021	16.533	0.820	1.130	0.020	16.245	0.850	1.079	0.021	16.029	0.850
% Lean	83.752	0.556	79.359	0.793	82.681	0.537	78.499	0.822	84.342	0.556	80.399	0.821
~6 weeks of age												
Body Mass, g	33.082	0.599	1.413	0.014	33.313	0.597	1.416	0.014	33.299	0.620	1.410	0.014
% Fat	1.106	0.023	17.001	0.877	1.133	0.023	16.276	0.877	1.075	0.024	16.896	0.908
% Lean	$3.4*10^{11}$	$1.6*10^{10}$	78.949	0.809	$3.2*10^{11}$	$1.6*10^{10}$	79.909	0.810	$3.6*10^{11}$	$1.6*10^{10}$	79.347	0.838
~7 weeks of age												
Body Mass, g	1.544	0.008	1.427	0.013	1.549	0.008	1.423	0.013	1.541	0.009	1.421	0.014
~8-9 weeks of age[a]												
Body Mass, g	38.802	0.697	1.436	0.014	38.194	0.695	1.446	0.014	38.461	0.721	1.434	0.014
% Fat	1.119	0.034	15.811	1.152	1.153	0.034	14.938	1.153	1.063	0.035	16.780	1.193
% Lean	$3.4*10^{9}$	$1.9*10^{8}$	78.520	1.012	$3.2*10^{9}$	$1.9*10^{8}$	79.469	1.012	$3.7*10^{9}$	$2.0*10^{8}$	78.489	1.048
Post exercise												
Body Mass, g	$8.3*10^{3}$	372.5	1.424	0.012	$8.4*10^{3}$	371.3	1.435	0.012	$8.0*10^{3}$	385.4	1.421	0.013
% Fat	0.948	0.043	3.272	0.118	1.010	0.043	3.185	0.118	0.900	0.044	3.232	0.122
% Lean	$4.4*10^{9}$	$2.2*10^{8}$	81.935	0.816	$4.1*10^{9}$	$2.2*10^{8}$	82.523	0.817	$4.7*10^{9}$	$2.3*10^{8}$	82.093	0.845
% change in mass	-4.576	1.193	-2.594	1.300	-2.877	1.151	-2.340	1.301	-6.441	1.193	-2.811	1.346
% change in % fat	-33.236	5.269	-32.140	5.137	-26.939	5.086	-27.199	5.139	-33.688	5.272	-37.515	5.320
% change in % lean	0.507	0.908	1.566	0.901	1.495	0.876	1.458	0.902	-1.656	0.908	1.624	0.933

[a]At ~8 weeks of age body composition measures were taken immediately prior to (in) and following (out) 6 days of wheel access. Percent body fat (and lean) was calculated as (fat mass/body mass)*100. Percent change variables were calculated as [(out - in)/in]*100.
